# Effect of Baoshenfang Formula on Podocyte Injury via Inhibiting the NOX-4/ROS/p38 Pathway in Diabetic Nephropathy

**DOI:** 10.1155/2019/2981705

**Published:** 2019-04-16

**Authors:** Fang-qiang Cui, Long Tang, Yan-bin Gao, Yue-fen Wang, Yuan Meng, Cun Shen, Zi-long Shen, Zhi-qiang Liu, Wen-jing Zhao, Wei Jing Liu

**Affiliations:** ^1^Department of Nephrology, Beijing Hospital of Traditional Chinese Medicine, Capital Medical University, 23 Meishuguanhou Street, Dongcheng District, Beijing 100010, China; ^2^Beijing Key Lab of TCM Collateral Disease Theory Research, No. 10, Youanmenwai, Xitoutiao, Fengtai District, Beijing 100069, China; ^3^School of Traditional Chinese Medicine, Capital Medical University, No. 10, Youanmenwai, Xitoutiao, Fengtai District, Beijing 100069, China; ^4^Key Laboratory of Chinese Internal Medicine of Ministry of Education and Beijing, Dongzhimen Hospital Affiliated to Beijing University of Chinese Medicine, Beijing 100700, China

## Abstract

Diabetic nephropathy (DN) is a serious kidney-related complication of type 1 and type 2 diabetes. The Chinese herbal formula Baoshenfang (BSF) shows therapeutic potential in attenuating oxidative stress and apoptosis in podocytes in DN. This study evaluated the effects of BSF on podocyte injury *in vivo* and *in vitro* and explored the possible involvement of the nicotinamide adenine dinucleotide phosphate-oxidase-4/reactive oxygen species- (NOX-4/ROS-) activated p38 pathway. In the identified compounds by mass spectrometry, some active constituents of BSF were reported to show antioxidative activity. In addition, we found that BSF significantly decreased 24-hour urinary protein, serum creatinine, and blood urea nitrogen in DN patients. BSF treatment increased the nephrin expression, alleviated oxidative cellular damage, and inhibited Bcl-2 family-associated podocyte apoptosis in high-glucose cultured podocytes and/or in diabetic rats. More importantly, BSF also decreased phospho-p38, while high glucose-mediated apoptosis was blocked by p38 mitogen-activated protein kinase inhibitor in cultured podocytes, indicating that the antiapoptotic effect of BSF is p38 pathway-dependent. High glucose-induced upexpression of NOX-4 was normalized by BSF, and NOX-4 siRNAs inhibited the phosphorylation of p38, suggesting that the activated p38 pathway is at least partially mediated by NOX-4. In conclusion, BSF can decrease proteinuria and protect podocytes from injury in DN, in part through inhibiting the NOX-4/ROS/p38 pathway.

## 1. Introduction

More and more studies have demonstrated that DN is the leading cause of the end stage of renal diseases [1]. Microalbuminuria is the typical clinical symptom of the early stage of DN. Meanwhile, microalbuminuria can induce renal dysfunction and lead to the rapid progression of DN [2]. Thus, decreasing urinary protein excretion has been an important therapeutic strategy for DN [3]. The compound formula of traditional Chinese medicine (TCM) has been widely used for the treatment of DN in China. However, the mechanism underlying a therapeutic effect has been unclear.

Podocyte injury is the key cause of proteinuria in DN [4, 5]. However, the molecular mechanism of podocyte injury in DN remains unclear. The previous study has demonstrated that increased ROS plays a key role in podocyte injury in DN [6, 7]. NOX-4, as an important number of nicotinamide adenine dinucleotide phosphate (NADPH) oxidase, is the major source of ROS production in podocyte [7]. Moreover, ROS can induce p38 phosphorylation to activate the p38 pathway, which is an important pathomechanism of podocyte injury in DN. Therefore, the NOX-4/ROS/p38 pathway has been the research focus in recent years [8, 9].

Baoshenfang (BSF), a kind of TCM compound, consists of a group of herbal medicines including Astragalus membranaceus, Salvia miltiorrhiza bunge, Fructus ligustri lucidi, leeches, and scorpions. BSF has been widely used in treating DN in our clinical practice. We found that BSF can significantly reduce proteinuria and delay progression of DN. More importantly, Astragaloside IV, a major active component of BSF, exhibits its antioxidant properties and antiapoptotic effects on podocytes in the treatment of DN in the previous study. However, the effect of BSF on podocyte injury, oxidative stress, and p38 pathway in DN has not been explored.

## 2. Methods

### 2.1. Clinical Trial

This trial has been approved by the Ethics Committee of Beijing traditional Chinese medicine Hospital (Approval NO16ZY06). The study is a randomized, controlled, and single-blind trial. Total 79 participants were collected for our trial. All participants should conform to the diagnostic criteria of diabetic kidney disease (DKD). Participants were randomly divided into control group and BSF group. Participants of two groups were given to basic therapy of DKD. Moreover, participants of the BSF group received BSF therapy. The intervention lasted for 12 weeks. The levels of HbA1c, serum lipid, serum creatinine, blood urea nitrogen, and 24 h urinary protein were detected at 0 and 12 weeks of intervention.

### 2.2. Animals

Our study was performed in accordance with the National Institutes of Health Guide. Sprague-Dawley (SD) male rats weighing 440 g to 460 g each were purchased from the Chinese Academy of Medical Sciences (Beijing, China). In order to induce diabetic rats, the rats were intraperitoneally injected streptozotocin (STZ, 60 mg/kg, Sigma, St. Louis, MO, USA) dissolved in 0.1 M citrate buffer (pH 4.5). The rats of the normal control (NC) group were intraperitoneally injected an equal volume of vehicle. The serum glucose was measured at 48 h after the injection of STZ. The rats of serum glucose ≥ 16.7 mmol/L were considered as diabetic rats. Our study consisted of three groups: normal control group (NC group), diabetes mellitus group (DM group), and Baoshenfang group (BSF group). Each group had 12 rats. In the BSF group, the rats were treated with BSF solution (BSF superfine powder, 0.75 g/kg/d, gavage). In the DM and NC groups, the rats were treated with an equal volume of vehicle (normal saline, gavage). All rats of three groups were treated for 12 weeks. At the end of 0, 4, 8, and 12 weeks, serum glucose and urinary albumin excretion (UAE) were measured. After that, the rats were killed and the renal cortex was collected for study purposes.

### 2.3. High-Performance Liquid Chromatography-Electrospray Ionization/Mass Spectrometer (HPLC-ESI/MS𝑛) Analysis

A Shimadzu UHPLC system (Kyoto, Japan) equipped with a LC-30AD solvent delivery system, a SIL-30AC autosampler, a CTO-30A column oven, a DGU-20A3 degasser, and a CBM-20A controller was used for HPLC-ESI/MSn analysis. The compounds were separated by a Waters ACQUITY UPLC HSS T3 (2.1 × 100 mm, 1.8 *μ*m) at 35°C. The flow rate of the mobile phase was 0.4 ml/min under a gradient program. The mobile phase consisted of 0.05% formic acid in water (A) and acetonitrile (B). The gradient system was 0-1 min, 2% B; 1–40 min, 2–50% B; 40–53 min, 50–95% B; 53–56 min, 95% B; 56-56.1 min, 95-2% B; and 56.1-60 min, 2% B. To monitor the peak intensity, the diode-array detector was set at 254 nm. The TripleTOF™ 5600+ system with a Duo Spray source (SCIEX, Foster City, CA, USA) was used for acquiring the mass spectra in negative and positive ESI mode. For TOF-MS and TOF-MS/MS analysis, the spectra covered the range from *m*/*z* 50 to 1,250 Da and 50-1250 Da. The data were analyzed by PeakView Software™ 2.2 (SCIEX, Foster City, CA, USA).

### 2.4. Preparation of Rat Serum-Containing Drug

Twenty healthy SD rats were randomly divided into the BSF group and control group. The rats of the BSF group were gavaged with BSF solution at a dose of 2 g/ml twice per day for three days. Rats of the NC group were gavaged with an equal amount of vehicle (normal saline) as normal control. Then, all rats were sacrificed and the serum was collected and isolated. The isolated serum was then removed in water bath for 30 min at 56°C. The serum was finally restored in the freezer at -70°C for further study.

### 2.5. Cell Culture and Treatment

The conditionally immortalized mouse podocyte line, which was obtained from the national platform of experimental cell resources for Sci-Tech, was used in our experiment. DMEM/low-glucose (HyClone) medium supplemented with 10% fetal bovine serum (Excell) was applied for cultured podocyte. In order to induce cell proliferation, the podocyte was cultured in medium with IFN-*γ* (PeproTech, Rocky Hill, New Jersey, USA) at 33°C. In order to induce cell differentiation, the podocyte was cultured in medium without IFN-*γ* at 37°C. The cultured podocyte was treated with normal rat serum for 24 h before the following study. The cultured podocyte was divided into five groups: NC group, HG group, SiNOX-4 group, p38 pathway inhibition group, and BSF group. The podocyte of the NC group was treated with DMEM containing 5.5 mmol/L glucose + 24.5 mmol/L mannitol. The podocyte of other groups was treated with DMEM containing 5.5 mmol/L glucose + 24.5 mmol/L glucose. The serum containing BSF was added to the medium of cultured podocyte in the BSF group. NOX-4 siRNA was transfected as described below. SB203580 (Santa Cruz, CA, USA) was added to the medium of the cultured podocyte for p38 pathway inhibition. After being treated for 72 h, the podocyte was collected for study purposes.

### 2.6. siRNA Transfection

NOX-4 siRNA was purchased from Santa Cruz Biotechnology (Santa Cruz, CA, USA). NOX-4 siRNA was transfected by the Lipofectamine 2000 transfection reagent (Invitrogen) according to the manufacturer's protocol. NOX-4 protein level was assayed by western blot for the confirmation of transfection. The transfection lasted for 48 h, and the podocyte was collected for experiments.

### 2.7. Western Blot Analysis

The renal cortex and collected podocyte were lysed using a lysis buffer on ice for 30 min. Protein was extracted from lysed tissues. The extracted protein was added to 10% SDS-PAGE and separated through electrophoresis. Protein was then transferred from SDS-PAGE to polyvinylidene difluoride membranes. After that, the membrane was moved to 5% nonfat dry milk in PBS + 0.05% Tween 20 and the blocked process was lasted for 1 h. The primary antibodies were then added to membranes and incubated at 4°C overnight. The membranes was washed by PBS and incubated with peroxidase secondary antibody for 1 h at room temperature. Antibodies and dilutions included the following: anti-nephrin antibody (Abcam, UK, Ab136894, 1 : 2000), anti-NOX-4 antibody (Abcam, UK, Ab109225, 1 : 1000), anti-p38 antibody (Abcam, UK, Ab31828, 1 : 1000), anti-p38 (phospho Y182) antibody (Abcam, UK, Ab47363, 1 : 1000), anti-Bax antibody (Abcam, UK, Ab7977, 1 : 500), anti-Bcl-2 antibody (Abcam, UK, Ab7973, 1 : 1000), and anti-GAPDH antibody (Proteintech, Chicago, IL, USA, 10494-1-AP, 1 : 1000). The blots were visualized with LumiGLO reagent and peroxide, followed by exposure to X-ray film. Western blot analyses were performed at least in triplicate.

### 2.8. Capillary Electrophoresis Immunoquantification

For quantitative capillary isoelectric immunoassay, the simple western immunoblots were performed on a Wes instrument (ProteinSimple) using the Size Separation Master Kit with Split Buffer (12–230 kDa) according to the manufacturer's standard instruction; 3 *μ*L of protein was run on the WES system using the following antibodies: anti-p38 antibody (Abcam, Cambridge, MA, USA), anti-p38 (phospho Y182) antibody (Abcam), and anti-GAPDH antibody (Proteintech, Chicago, IL, USA). All other reagents (antibody diluent, secondary antibodies) were from ProteinSimple. The Compass software (ProteinSimple version 3.1.8) was used to program the Wes instrument and for the quantification of the western Immunoblots. Output data was displayed from the software-calculated average of nine exposures (1–512 s). Run conditions were as recommended by the manufacturer. Peak areas were determined using Compass software and normalized to anti-GAPDH.

### 2.9. Real-Time RT-PCR

The TRIzol reagent (Invitrogen, Carlsbad, CA, USA) was added to the renal cortex and cultured podocyte, and total RNA was isolated from the renal cortex and cultured podocyte. After that, the superscript RT kit (Invitrogen) was used to reverse-transcribe from RNA into cDNAs. The level of RNA was calculated by the 2^-△△Ct^method. The sequences of primers are the following: mouse nephrin: forward primer, CCCAACACTGGAAGAGGTGT-3, reverse primer, CTGGTCGTAGATTCCCCTTG; mouse Nox-4: forward primer, CAGAGACATCCAATCATTCCAGTG, reverse primer, CTGGATGTTCACAAAGTCAGGTCT; mouse Bax: forward primer, CAGGGTTTCATCCAGGATCGAGCAGG, reverse primer, CGGGGGG-AGTCCGTGTCCACGTCAG; mouse Bcl-2: forward primer: CCAGCG-TGTGTGTGCAAGTGTAAAT, reverse primer, ATGTCAATCCG-TAGGAATCCCAACC; rat nephrin: forward primer, GCAAAGACTGGAAGAGGTGT, reverse primer, CTGGCCG-TAGATTCCCAGTG. RT-PCR analyses were performed at least in triplicate.

### 2.10. Immunofluorescence

When cells were grown to 80% confluences, 4% paraformaldehyde was added to cells for 30 min. After being blocked by 2.5% normal serum, the podocyte was incubated with anti-p38 (phospho Y182) antibody (Abcam) at 4°C overnight. After washing by PBS, cells were incubated with Alexa Fluor® 594 donkey anti-rabbit IgG (Invitrogen) at room temperature for 2 h. The podocyte was then counterstained by DAPI for 5 min. Cells were observed under a confocal microscope (Leica TCS SP5 MP, Leica, Heidelberg, Germany).

### 2.11. Flow Cytometry Analysis

To collect podocytes, cells of three groups were centrifugated at 2000 rpm for 5 min. The collected cells were then washed twice with PBS and resuspended with binding buffer. After that, Annexin V-FITC and PI were added and the podocytes were collected after 15 min. The data of apoptotic cells was analyzed using FACScan. Flow cytometry analyses of different groups were performed at least in triplicate.

### 2.12. Determination of ROS

ROS detection kit (Beyotime, Haimen, China) was used for ROS measurement according to the manufacturer's protocol in our study. The podocyte was cultured in a 24-well plate and treated with a different cultured medium, and then the podocyte was incubated with 10 *μ*M DCFH-DA for 20 min at 37°C. The fluorescence intensity was observed and recorded by a fluorescent microscope.

### 2.13. Caspase-3 Activity

Caspase-3 kit (Beyotime, Haimen, China) was used for caspase-3 activity detection. Podocyte was cultured in a 6-well plate. The podocyte was treated with a different cultured medium for 24 h. After that, the podocyte was collected and lysed with a lysis buffer on ice. The reaction buffer (80 *μ*L) and caspase-3 substrate (10 *μ*L) were added to the cell lysate (10 *μ*L). The optical density (OD) was assayed by a spectrophotometer at 405 nm.

### 2.14. Statistical Analysis

Data were presented as mean ± S.E.M., counts, or percentage. Statistical analyses were performed by one-way ANOVA followed by the Bonferroni multiple comparison test (for comparison of more than 2 groups) or Student's *t*-test (for comparison of 2 groups) unless otherwise stated. *P* < 0.05 was considered statistically significant.

## 3. Results

### 3.1. Characteristics of Pure Compounds in BSF

**Figure 1:**
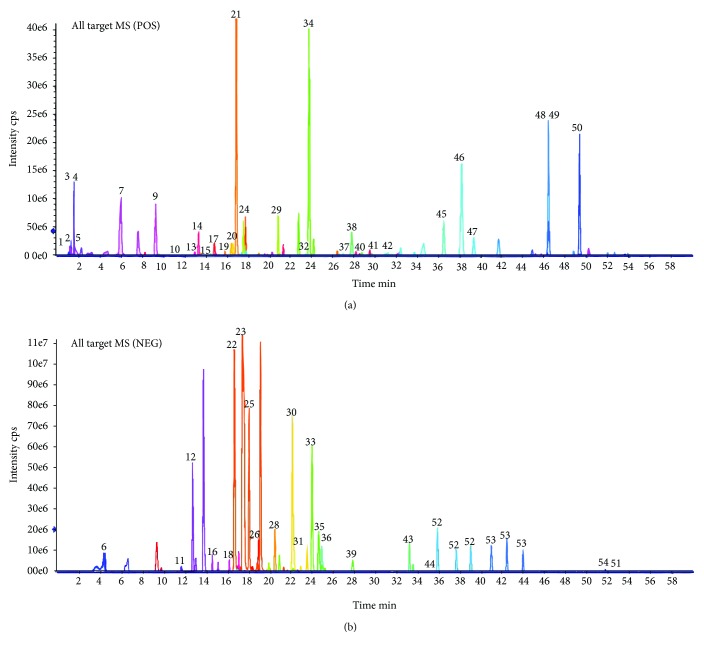
Ion chromatograms of BSF analyzed by high-performance liquid chromatography-electrospray ionization/mass spectrometry (HPLC-ESI/MS𝑛) analysis. (a) Positive base peak MS spectrum. (b) Negative base peak mass spectrometry (MS) spectrum.

Liquid chromatography-mass spectrometry (LC-MS) has been a common way of substance analysis of TCM in recent years. In our study, substance analysis for BSF was performed in both negative and positive ESI modes. The MS spectrum of the negative base peak and positive base peak is displayed in Figure 1. There were 54 kinds of substances of BSF identified by MS. The identified compound is displayed in Table 1, including flavones, coumarins, phenylethanoid glycosides, phenolic acids, saponins, and organic acids. More importantly, many substances such as quercetin (number 36), salvianolic acid B (number 30), luteolin (number 35), and astragaloside IV (number 43) might offer the biological activity of antioxidative stress. Therefore, BSF's antioxidative activity and the associated mechanism were studied in our research.

### 3.2. BSF Decreased 24 h Urinary Protein and Improved Renal Function in Patients with DKD

In our clinical trial, 79 participants of DKD patients were collected for our research. All participants were randomly divided into control group and BSF group. The age, sex, serum albumin, blood urea nitrogen, and 24 h urinary protein were not significantly different before treatment between control group and BSF groups. After intervention for 12 weeks, the 24 h urinary protein of BSF group was significantly decreased compared with the control group. Meanwhile, the levels of serum creatinine and blood urea nitrogen in the BSF group were lower than those in the control group (Table 2).

### 3.3. BSF Ameliorated Proteinuria and Improved Renal Function in Diabetic Rats

**Figure 2:**
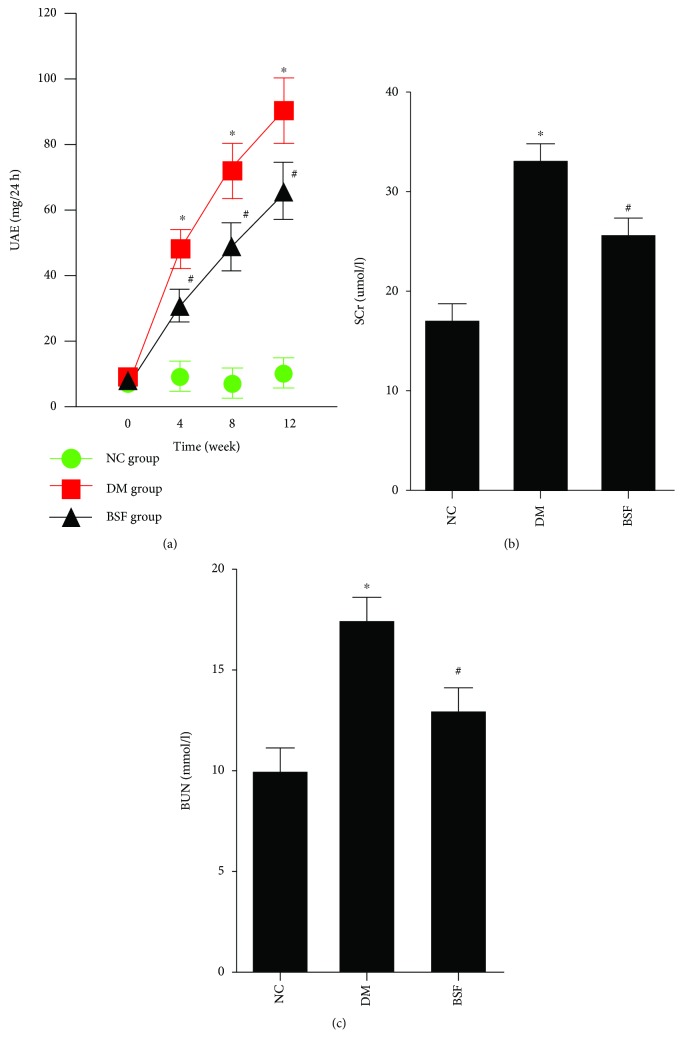
Effect of BSF on urinary albumin excretion (UAE), serum creatinine (SCr), and blood urea nitrogen (BUN) in diabetic rats. The levels of UAE (a), SCr (b), and BUN (c) in different groups. All the indicators were increased in DM rats, but lowered by BSF treatment. ^∗^*P* < 0.05 versus normal control (NC), ^#^*P* < 0.05 versus DM.

Next, urinary albumin excretion, serum creatinine, and blood urea nitrogen were measured in different rodent groups. Our results showed that urinary albumin excretion of the DM group was significantly increased at 4 weeks and gradually further enhanced at 8 and 12 weeks compared with the NC group. Urinary albumin excretion of the BSF group was significantly decreased compared with the DM group at 4, 8, and 12 weeks. Moreover, serum creatinine and blood urea nitrogen were significantly increased in the DM group compared with the NC group at 12 weeks. However, the levels of serum creatinine and blood urea nitrogen were significantly decreased in the BSF group compared with the DM group (Figure 2).

### 3.4. BSF Increased the Nephrin Expression in High Glucose Cultured Podocyte and Diabetic Rats

**Figure 3:**
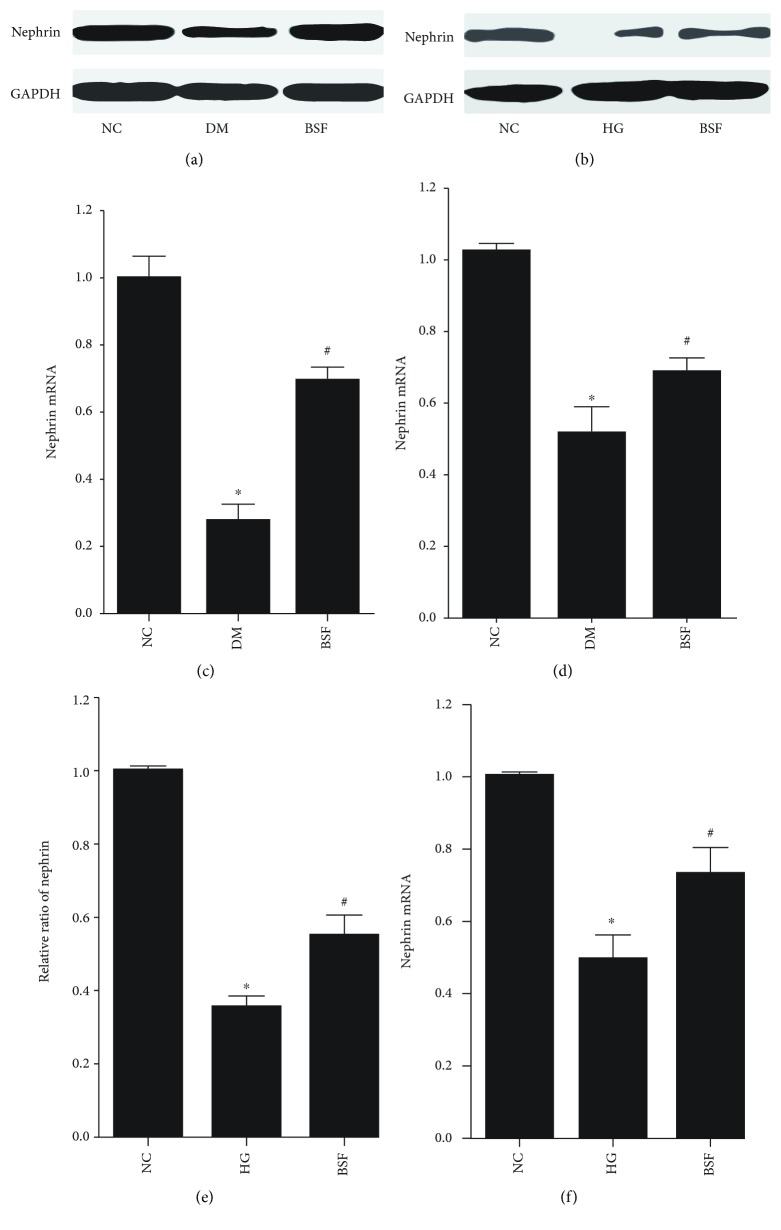
Effect of BSF on nephrin expression in podocyte in vivo and in vitro. (a) Representative band of nephrin protein in the cortex of rats. (b) Representative band of nephrin protein in cultured podocyte. (c) Comparison of the grey values of nephrin protein in podocyte of rats. (d) Comparison of mRNA levels of nephrin in podocyte of rats. (e) Comparison of the grey values of nephrin protein in cultured podocyte. (f) Comparison of mRNA levels of nephrin in cultured podocyte. Nephrin mRNA or protein level was significantly decreased in diabetic rats and high glucose (HG) cultured podocytes. BSF treatment significantly increased nephrin expression. ^∗^*P* < 0.05, ^∗∗^*P* < 0.01 versus normal control (NC), and ^#^*P* < 0.05 versus DM or HG.

Nephrin is a signature molecule of podocyte, and decreased nephrin expression is a hallmark of podocyte injury. Therefore, nephrin expression of different groups was subsequently studied. Nephrin protein and mRNA expressions were quantified by WB and RT-PCR, respectively. We found that either protein or mRNA level of nephrin was significantly decreased in the DM group compared with the NC group. Interestingly, the expression of nephrin protein or mRNA was significantly increased in the BSF group compared with the DM group (Figures 3(a), 3(c), and 3(d)). And similar results were obtained in an *in vitro* study (Figures 3(b), 3(e), and 3(f)).

### 3.5. BSF Decreased Cellular Apoptosis in Diabetic Rats and High Glucose Cultured Podocytes

**Figure 4:**
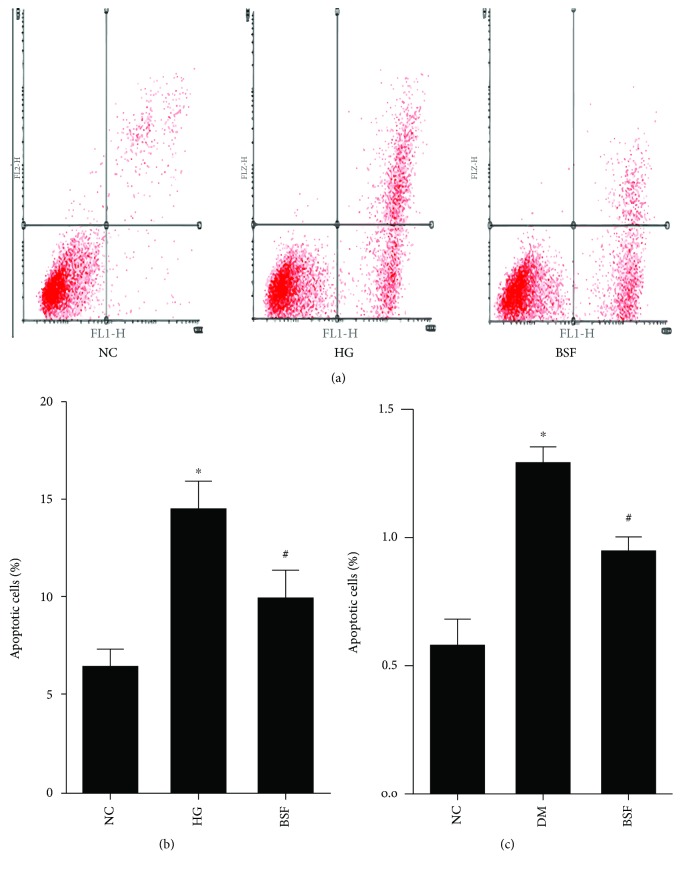
Effect of BSF on podocyte apoptosis in vivo and in vitro. (a) Representative photograph of flow cytometry analysis in vitro. (b) Comparison of apoptotic cells in cultured podocyte of different groups evaluated by flow cytometry. (c) Comparison of apoptotic index in glomerulus of different groups assayed by TUNEL. BSF treatment significantly decreased high glucose (HG) or hyperglycemia-induced cellular apoptosis. ^∗^*P* < 0.05 versus normal control (NC) and ^#^*P* < 0.05 versus DM or HG.

Subsequently, early and late apoptosis was subsequently evaluated by flow cytometry after coupled staining with FITC Annexin V in cultured podocytes. Either early or late podocyte apoptosis was higher in the high glucose group than in normal control. Such alteration was significantly inhibited by the serum with BSF (Figures 4(a) and 4(b)). Similarly, hyperglycemia induced an increase in cellular apoptosis in glomerulus, which was reduced by BSF treatment (Figure 4(c)).

### 3.6. BSF Inhibited NOX-4-Mediated Oxidative Stress in High Glucose Cultured Podocyte

**Figure 5:**
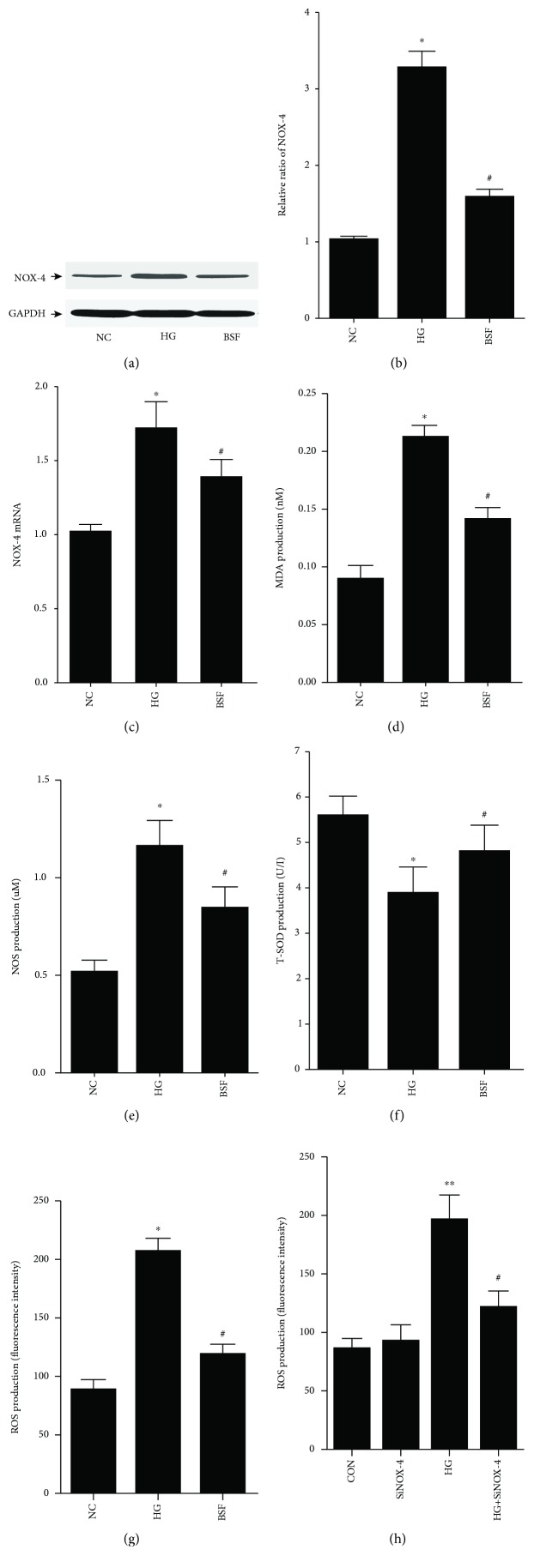
Effect of BSF on NOX-4-mediated oxidative stress in cultured podocyte. (a) Representative band of NOX-4 protein in cultured podocyte. (b) Comparison of the grey values of NOX-4 protein in cultured podocyte. (c) Comparison of mRNA levels of NOX-4 in cultured podocyte. BSF suppressed the upexpression of NOX-4 after exposure of podocyte to high glucose (HG). (d–f) Comparison of MDA, NOS, and T-SOD levels measured by ELISA in cultured podocyte. (g, h) Comparison of ROS production in cultured podocyte. High glucose induced a marked enhancement of MDA, NOS, and ROS levels, a significant suppression of T-SOD level. Compared with the HG group, MDA, NOS, and ROS levels were significantly decreased, while the T-SOD level was significantly increased in the BSF group. And the upregulated ROS production was suppressed by NOX-4 silencing. ^∗^*P* < 0.05, ^∗∗^*P* < 0.01 versus normal control (NC), ^#^*P* < 0.05, and ^##^*P* < 0.01 versus HG.

NOX-4, the main NADPH oxidase isoform contributing to the increased oxidative stress, was evaluated in vitro. We found that NOX-4 protein or mRNA level was indeed increased in high glucose-treated podocytes, but suppressed by BSF (Figures 5(a)–5(c)). In addition, high glucose induced a marked enhancement of MDA, NOS, and ROS levels, a significant suppression of the T-SOD level. Compared with the HG group, MDA, NOS, and ROS levels were significantly decreased, while the T-SOD level was significantly increased in the BSF group (Figures 5(d)–5(g)). Moreover, the upregulated ROS level was significantly reduced by NOX-4 silencing (Figure 5(h)), suggesting that the antioxidation effect is NOX-4 blockage dependent.

### 3.7. BSF Inhibited Bcl-2 Family-Associated Apoptosis by Inactivating the p38 Pathway in High Glucose Cultured Podocyte

**Figure 6:**
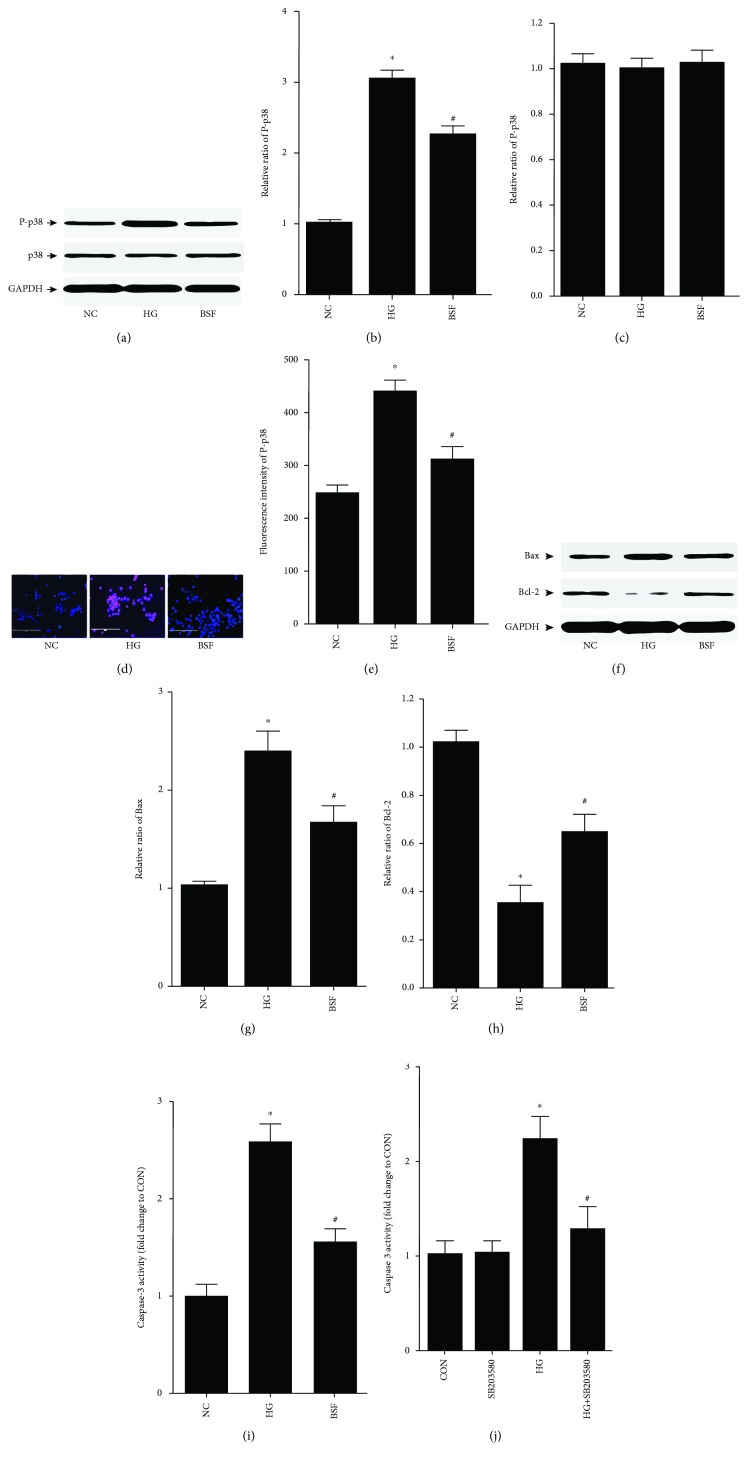
Effect of BSF on p38 pathway-induced apoptosis in HG cultured podocytes. (a) Representative band of p38 protein and P-p38 protein in cultured podocyte. (b, c) Comparison of the grey values of p38 and P-p38 protein in cultured podocytes. P-p38, but not p38 protein expression, was significantly increased in the HG group compared with the NC group. BSF decreased the expression of P-p38 protein. (d) Representative photograph of P-p38 staining (red) and cell nucleus (DAPI blue) in cultured podocyte. (e) Comparison of the fluorescence intensity of P-p38 protein in cultured podocyte. BSF significantly decreased the fluorescence intensity of P-p38 compared with the HG group. (f) Representative band of Bax protein and Bcl-2 protein in cultured podocyte. (g, h) Comparison of the grey values of Bax protein in cultured podocyte. HG upregulated the expression of Bax protein and downregulated the expression of Bcl-2 protein, which was reversed by BSF. (i, k) Comparison of caspase-3 activity in cultured podocyte. Either BSF or SB203580 could decrease the high caspase-3 activity induced by HG. ^∗^*P* < 0.05 versus NG. ^#^*P* < 0.05 versus HG.

To explore the effect of BSF on the p38 pathway in podocyte, p38 and/or phospho-p38 (P-p38) protein expression was assessed by western blot and immunofluorescence. Western blot analysis showed that p38 protein expression had no significant difference among different groups. However, the P-p38 protein level was significantly increased in the HG group. Interestingly, the high expression of P-p38 was also downregulated by BSF in an in vitro study (Figures 6(a)–6(c)). Moreover, the results of immunofluorescence indicated that HG promoted P-p38 protein nuclear translation, which was inhibited by BSF therapy (Figures 6(d) and 6(e)). The antiapoptotic protein Bcl-2 and proapoptotic protein Bax in the Bcl-2 family were then studied. Our results showed that Bax was significantly increased and Bcl-2 was significantly decreased in the HG group. BSF decreased Bax expression and increased Bcl-2 expression induced by HG in an in vitro study (Figures 6(f)–6(h)). Accordingly, HG-induced high caspase-3 activity was lowered by BSF treatment (Figure 6(i)). More importantly, we also found that the antiapoptotic effect of BSF was p38 pathway-dependent, since p38 pathway inhibitor SB203580 could suppress the podocyte apoptosis induced by HG (Figure 6(k)).

### 3.8. p38 Phosphorylation Induced Podocyte Apoptosis Was NOX-4-Dependent

**Figure 7:**
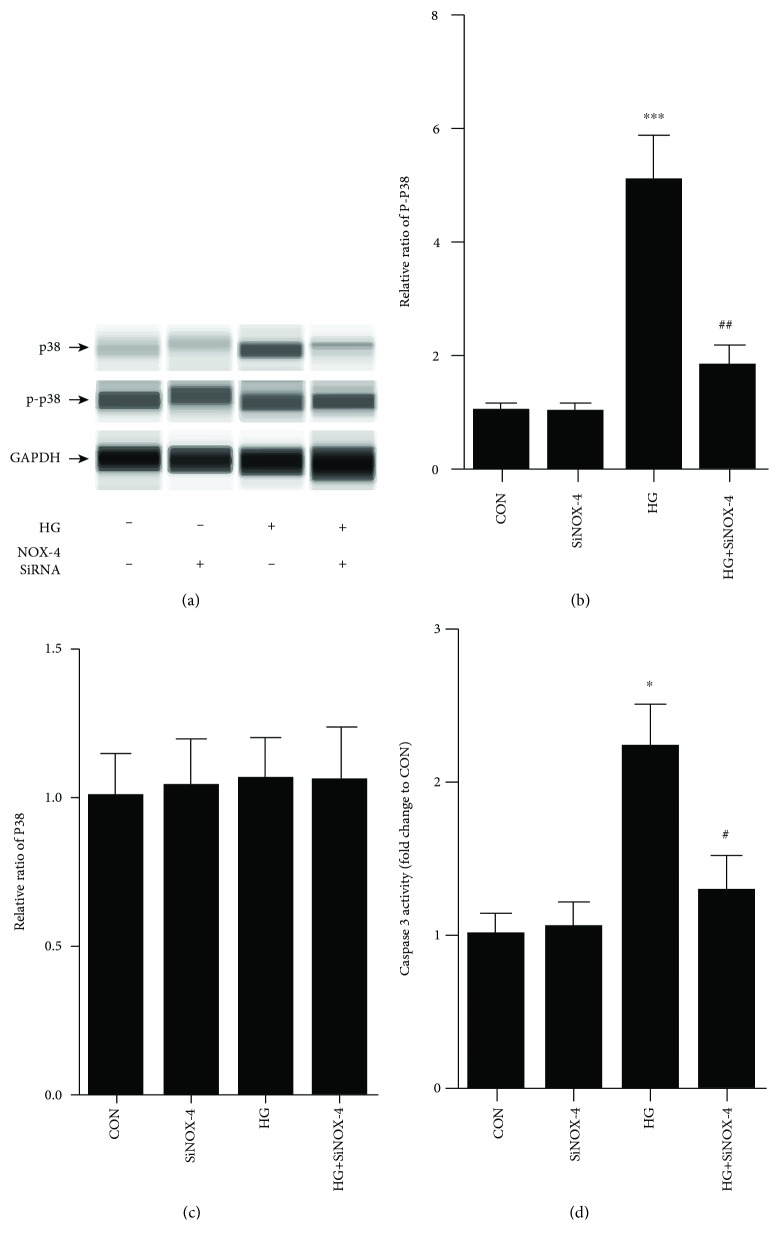
Effect of NOX-4 siRNA on p38 pathway-induced apoptosis in HG cultured podocytes. (a) Representative band of p38 and P-p38 protein in cultured podocyte. (b, c) Comparison of the grey values of P-p38 and p38 protein in cultured podocyte. NOX-4 siRNA significantly decreased the expression P-p38 protein compared with the HG group. (d) Comparison of caspase-3 activity in cultured podocyte. NOX-4 siRNA significantly decreased the caspase-3 activity in HG cultured podocytes. ^∗^*P* < 0.05 and ^∗∗∗^*P* < 0.001 versus NG. ^#^*P* < 0.05 and ^##^*P* < 0.01 versus HG.

To demonstrate the relationship between p38 phosphorylation and NOX-4 activation, the p38 pathway was studied after the podocytes were transfected with NOX-4 siRNA. In our study, NOX-4 silence significantly inhibited p38 phosphorylation induced by HG in cultured podocytes (Figures 7(a)–7(c)). Moreover, the high caspase-3 activity was significantly decreased by NOX-4 siRNA in HG-cultured podocytes (Figure 7(d)). It indicates that the antiapoptotic effect of BSF is NOX-4/p38 pathway-dependent.

## 4. Discussion

Diabetic nephropathy, as the most common microvascular complication of diabetic mellitus, has been the leading cause of end-stage renal disease [2]. Data from clinical trials indicates that 40% of end-stage renal disease patients and 50% of dialysis and kidney transplant patients are induced by DN [10]. However, the pathomechanism of DN has been unclear. Meanwhile, the effective clinical intervention is limited for DN patients. Thus, it is important for us to seek a potential therapeutic target of DN. As we know, microalbuminuria is the typical clinical symptom of the early stage of DN [11]. Microalbuminuria is also the key cause which can lead to rapid progression of DN [1]. Moreover, reducing urinary protein excretion has been the important therapeutic method in recent years. BSF, a kind of traditional Chinese medicine compound, has been widely used in treatment of DN in our clinical practice. BSF can significantly decrease proteinuria and serum creatinine of DN patients, although it has no obvious influence on eGFR (data was not shown). In our in vivo study, we found that BSF significantly decreased urinary albumin excretion of diabetic rats. Moreover, in the main substances of BSF, it has been demonstrated that quercetin [12], salvianolic acid B [13], and luteolin [14] can decrease oxidative stress in many diseases. Moreover, astragaloside IV could protect podocytes from injury via inhibiting oxidative stress, as demonstrated in a previous study [15]. It indicates that BSF probably prevents the progression of DN by offering antioxidative activity.

Podocyte, as an important component of glomerular filtration membrane, has been the research focus and important potential therapeutic target of DN in recent years [16–18]. Meanwhile, podocyte injury plays a key role in increased urinary albumin in DN [19–22]. Nephrin, a signature molecule of podocyte, can regulate many pivotal functions of podocyte [5]. Research suggests that nephrin expression is important for signal transduction and cytoskeletal reorganization of podocyte [5]. Moreover, podocyte is a kind of terminal differentiation cell and has lost the ability for regenerative reparation. A decrease in the number of podocytes can lead to destruction of GBM and increased urinary albumin [23–25]. The previous study has demonstrated that podocyte apoptosis is the leading cause of the decrease in the number of podocytes in DN [19, 20]. Thus, decreased nephrin expression and increased podocyte apoptosis is a good marker of podocyte injury. Evidence suggests that nephrin expression is significantly decreased and podocyte apoptosis is significantly increased in DN. We also explored nephrin expression and podocyte apoptosis in diabetic rats and high glucose cultured podocyte in our study. In accordance with the previous study, nephrin expression is significantly decreased and podocyte apoptosis is significantly increased in DN. More importantly, BSF significantly increased nephrin expression and decreased podocyte apoptosis in diabetic rats and high glucose cultured podocytes. Our results indicate that BSF can protect podocytes from injury in DN.

Oxidative stress has been the central pathomechanism of podocyte injury in DN [7, 26]. ROS production is significantly increased in podocyte in DN, which has been demonstrated by many previous studies. Moreover, there are many ways involved in increased ROS production in podocyte. However, NADPH is the major source of ROS in podocyte in DN. NOX-4, as a member of the NADPH oxidase family, has been the key oxidase in increased ROS production in podocyte in DN [27]. It has been demonstrated that HG can increase NOX-4 expression and ROS production in podocyte, which is the major cause of podocyte apoptosis [7, 27]. Moreover, NOS, T-SOD, and MDA are also good markers of oxidative stress [15]. In order to explore the effect of BSF on podocyte oxidative stress, ROS, NOX-4, NOS, T-SOD, and MDA were detected in our in vivo and in vitro study. Our results showed that BSF significantly inhibited podocyte oxidative stress in DN.

The p38 pathway is an important mechanism of podocyte injury in DN [28]. Some reports suggest that the p38 pathway can be activated by oxidative stress in podocyte [9]. The p38 pathway has an intensive relationship with podocyte apoptosis [8]. The activated p38 pathway can lead to increased Bax expression and decreased Bcl-2 expression [29]. As we know, Bax is a kind of proapoptotic protein and Bcl-2 is a kind of inhibitor of apoptosis protein. Thus, increased podocyte apoptosis can be induced by the activated p38 pathway. Moreover, p38 pathway activation decreases nephrin expression and induces podocyte injury, which has been demonstrated by previous studies. To explore the molecular mechanism of BSF on podocyte injury, p38 pathway, Bax, and Bcl-2 expression were detected in our study. Our results showed that P-p38 protein was significantly increased in podocyte of the HG group. Moreover, Bax protein was significantly increased and Bcl-2 protein was significantly decreased in podocyte of the HG group. More importantly, BSF could significantly decrease p38 phosphorylation after exposure of podocyte to HG. Bax expression was significantly decreased, and Bcl-2 expression was significantly increased in the BSF group compared with the HG group.

**Figure 8:**
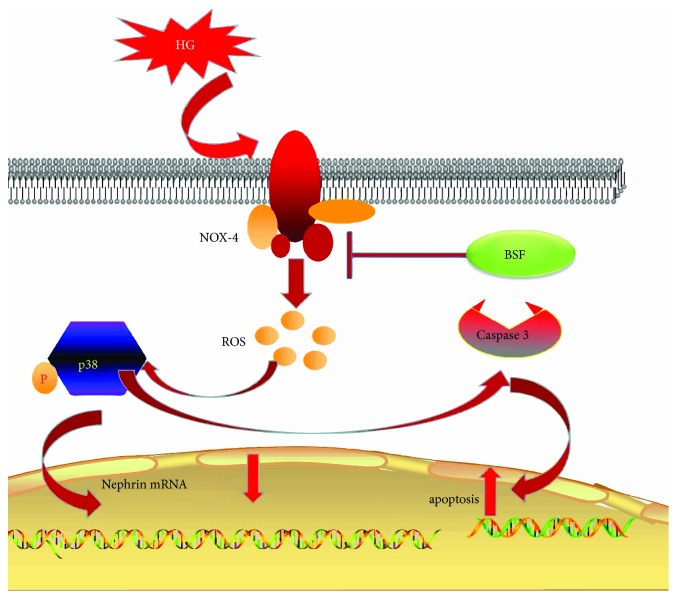
Schematic representation of the mechanisms of the NOX-4/ROS/p38 pathway in podocyte injury in high glucose condition. HG significantly increases NOX-4 expression in podocyte. And NOX-4-induced ROS overproduction triggers cascade phosphorylation reaction of the p38 pathway. Accordingly, activation of the p38 pathway can decrease nephrin expression and induce podocyte apoptosis. Baoshenfang formula might protect podocyte from HG-induced injury via inhibiting the NOX-4/ROS/p38 pathway.

In conclusion, BSF could prevent the development of DN. The renoprotection might be attributed to an inhibitive effect of the NOX-4/ROS/p38 pathway in podocytes (Figure 8).

## Figures and Tables

**Table 1:** Chemical components of BSF identified by HPLC-ESI/MS𝑛.

No.	Retention time	Formula	Adduct/charge	Experimental mass	Identification
1	1.18	C4H9NO3	[M + H]+	120.0655	Threonine
2	1.39	C7H13NO2	[M + H]+	144.1019	Stachydrine
3	1.63	C5H5N5	[M + H]+	136.0618	Adenine
4	1.65	C5H4N4O	[M + H]+	137.0458	6-Hydroxypurine
5	1.77	C6H13NO2	[M + H]+	132.1019	Leucine
6	4.44	C7H6O5	[M-H]-	169.0142	Gallic acid
7	6.04	C6H6O3	[M + H]+	127.039	5-Hydroxymethylfurfural
8	6.65	C9H10O5	[M-H]-	197.0455	Danshensu
9	9.35	C14H20O7.NH3	[M + H]+	318.1547	Salidroside
10	11.22	C17H24O9.NH3	[M + H]+	390.1759	Syringin
11	11.7	C9H6O4	[M-H]-	177.0193	Esculetin
12	12.78	C17H26O10.HCOOH	[M-H]-	435.1508	Loganin
13	13.03	C21H20O9	[M + H]+	417.118	Puerarin
14	13.39	C16H22O9	[M + H]+	359.1337	Sweroside
15	13.97	C10H8O5	[M + H]+	209.0444	Fraxetin
16	14.61	C35H46O20	[M-H]-	785.251	Echinacoside
17	14.88	C23H28O11.NH3	[M + H]+	498.197	Paeoniflorin
18	16.22	C36H48O20	[M-H]-	799.2666	
19	16.49	C10H8O4	[M + H]+	193.0495	Isoscopoletin
20	16.61	C10H10O4	[M + H]+	195.0652	Ferulic acid
21	16.96	C22H22O10	[M + H]+	447.1286	Calycosin-7-o-glucoside
22	17.11	C34H44O19	[M-H]-	755.2404	Forsythoside B
23	17.32	C21H20O12	[M-H]-	463.0882	Isoquercitrin
24	17.82	C21H20O11	[M + H]+	449.1078	Homoorientin (isoorientin)
25	18.38	C7H6O3	[M-H]-	137.0244	Salicylic acid
26	18.98	C20H18O10	[M-H]-	417.0827	Salvianolic acid D
27	19.16	C29H36O15	[M-H]-	623.1981	
28	20.52	C18H16O8	[M-H]-	359.0772	Rosmarinic acid
29	20.89	C21H18O12	[M + H]+	463.0871	Scutellarin
30	22.17	C36H30O16	[M-H]-	717.1461	Salvianolic acid B
31	22.24	C21H18O11	[M-H]-	445.0776	Baicalin
32	23.49	C15H12O4	[M + H]+	257.0808	Isoliquiritigenin
33	24.02	C26H22O10	[M-H]-	493.114	Salvianolic acid A
34	24.23	C27H34O11	[M + NH4]+	552.2439	Arctiin
35	24.64	C15H10O6	[M-H]-	285.0405	Luteolin
36	24.66	C15H10O7	[M-H]-	301.0354	Quercetin
37	27.4	C15H12O5	[M + H]+	273.0757	Naringenin
38	27.7	C30H32O12.NH3	[M + H]+	602.2232	Benzoylpaeoniflorin
39	27.85	C15H10O5	[M-H]-	269.0455	Apigenin
40	28.79	C16H12O6	[M + H]+	301.0707	Diosmetin
41	29.73	C15H10O5	[M + H]+	271.0601	Baicalein
42	31	C15H12O4	[M + H]+	257.0808	Liquiritigenin
43	33.2	C41H68O14	[M + FA-H]-	829.4591	Astragaloside IV
44	35.37	C16H12O5	[M-H]-	283.0612	Wogonin
45	36.5	C21H22O8	[M + H]+	403.1387	Nobiletin
46	38.17	C15H20O3	[M + H]+	249.1485	Parthenolide
47	39.32	C20H20O7	[M + H]+	373.1282	Tangeretin
48	46.36	C19H20O3	[M + H]+	297.1485	Cryptotanshinone
49	46.37	C18H12O3	[M + H]+	277.0859	Tanshinone I
50	49.29	C19H18O3	[M + H]+	295.1329	Tanshinone IIA
51	52.72	C18H32O2	[M-H]-	279.233	Linoleic acid
52	35.8/37.6/38.98	C43H70O15.HCOOH	[M-H]-	871.4697	Astragaloside II
53	40.9/42.4/43.9	C45H72O16.HCOOH	[M-H]-	913.4802	Astragaloside I
54	51.7/52.1/56.8/57.4	C30H48O3	[M-H]-	455.3531	Oleanolic acid

**Table 2:** Effect of BSF on biochemical indexes of DKD.

Groups	Control	BSF	*P* value
Sample size	39	40	—
Female sex (%)^a^	20 (51.3%)	18 (45.0%)	>0.05
Age (years)^b^	62.95 ± 7.24	63.35 ± 8.02	>0.05
HbA1c (%)^b^ (week 0)	7.81 ± 1.67	7.96 ± 1.40	>0.05
HbA1c (%)^b^ (week 12)	6.47 ± 1.92	6.46 ± 0.74	>0.05
Total cholesterol (mmol/l)^b^ (week 0)	6.04 ± 1.45	5.87 ± 1.14	>0.05
Total cholesterol (mmol/l)^b^ (week 12)	4.59 ± 0.90	4.44 ± 0.87	>0.05
Triglyceride (mmol/l)^b^ (week 0)	2.31 ± 0.89	2.29 ± 0.93	>0.05
Triglyceride (mmol/l)^b^ (week 12)	1.64 ± 0.45	1.54 ± 0.38	>0.05
Serum albumin (g/l)b (week 0)	28.6 ± 1.4	28.3 ± 1.7	>0.05
Serum albumin (g/l)^b^ (week 12)	33.2 ± 2.7	38.0 ± 2.4	<0.05
Serum creatinine (*μ*mol/l)^b^ (week 0)	121.6 ± 10.1	124.6 ± 13.0	>0.05
Serum creatinine (*μ*mol/l)^b^ (week 12)	115.6 ± 10.1	94.9 ± 13.0	<0.05
Blood urea nitrogen (mmol/l)^b^ (week 0)	7.5 ± 0.4	7.6 ± 0.7	>0.05
Blood urea nitrogen (mmol/l)^b^ (week 12)	6.7 ± 0.5	5.6 ± 0.3	<0.05
24 h urinary protein (g)^b^ (week 0)	3.4 ± 1.9	3.48 ± 2.1	>0.05
24 h urinary protein (g)^b^ (week 12)	3.18 ± 1.94	2.55 ± 1.67	<0.05

^a^The data are expressed as counts (%), and the *P* value for the between-group difference is calculated from the Wilcoxon rank sum test. ^b^The data are expressed as the mean ± S.E.M., and the *P* value for the two group comparisons is calculated using an independent-sample *t*-test.

## Data Availability

The data used to support the findings of this study are available from the corresponding author upon request.
